# Fractal Analysis on Machined Surface Morphologies of Soft-Brittle KDP Crystals Processed by Micro Ball-End Milling

**DOI:** 10.3390/ma16051782

**Published:** 2023-02-21

**Authors:** Qi Liu, Jian Cheng, Zhirong Liao, Mingyu Liu, Mingjun Chen, Linjie Zhao, Hongqin Lei, Wenyu Ding

**Affiliations:** 1 State Key Laboratory of Robotics and System, Harbin Institute of Technology, Harbin 150001, China; 2 Centre for Precision Manufacturing, Department of Design Manufacturing & Engineering Management (DMEM), University of Strathclyde, Glasgow G1 1XJ, UK; 3 Department of Mechanical, Materials and Manufacturing Engineering, University of Nottingham, Nottingham NG8 1BB, UK; 4 School of Engineering, College of Science, University of Lincoln, Lincoln LN6 7TS, UK

**Keywords:** KDP crystal, fractal dimension, box-counting approach, brittle-to-ductile transition, surface morphology analysis, material removal modes

## Abstract

The micro-defects on KH_2_PO_4_ (KDP) optic surfaces are mainly repaired by the micro-milling technique, while it is very easy to introduce brittle cracks on repaired surfaces, as KDP is soft and brittle. To estimate machined surface morphologies, the conventional method is surface roughness, but it fails to distinguish ductile-regime machining from brittle-regime machining directly. To achieve this objective, it is of great significance to explore new evaluation methods to further characterize machined surface morphologies. In this study, the fractal dimension (*F*_D_) was introduced to characterize the surface morphologies of soft-brittle KDP crystals machined by micro bell-end milling. The 3D and 2D fractal dimensions of the machined surfaces and their typical cross-sectional contours have been calculated, respectively, based on Box-counting methods, and were further discussed comprehensively by combining the analysis of surface quality and textures. The 3D *F*_D_ is identified to have a negative correlation with surface roughness (*S*_a_ and *S*_q_), meaning the worse the surface quality the smaller the *F*_D_. The circumferential 2D *F*_D_ could quantitively characterize the anisotropy of micro-milled surfaces, which could not be analyzed by surface roughness. Normally, there is obvious symmetry of 2D *F*_D_ and anisotropy on the micro ball-end milled surfaces generated by ductile-regime machining. However, once the 2D *F*_D_ is distributed asymmetrically and the anisotropy becomes weaker, the assessed surface contours would be occupied by brittle cracks and fractures, and corresponding machining processes will be in a brittle regime. This fractal analysis would facilitate the accurate and efficient evaluation of the repaired KDP optics by micro-milling.

## 1. Introduction

Potassium dihydrogen phosphate (KDP) crystals, known as an excellent non-linear optical material [1,2], have been widely adopted to generate harmonics for Nd: YAG laser systems [3,4]. While working under continuous laser irradiation, laser-induced damage defects (e.g., micro-cracks and micro pits [5,6,7]) are vulnerable to generate on the KDP surfaces, thus restricting the sustainable operations of inertial confinement fusion (ICF). In order to recycle and use these valuable KDP optics, the most cost-efficient way is to repair the damaged defects as quickly as possible to avoid them further growing to scrap the entire optics. Currently, the most flexible method is micro ball-end milling to achieve this objective [8,9]. However, there are some important issues that urgently need in-depth investigation before performing this micro-milling repair approach into the actual engineering application in ICF facilities [10]. One challenge is that as KDP optics are soft and brittle [11,12,13], brittle cracks are quite easily involved in micro-milling processes, deteriorating the machined surfaces, which might shorten the service performance and life of the repaired KDP optics [8,14,15]. Thus, it is significant to carry out a comprehensive evaluation of the repaired KDP surface morphologies.

The surface morphologies of the machined parts have a direct and significant effect on their ultimate service performance [16,17,18], especially for the optics components working with high-power lasers like KDP crystal [4,19,20,21]. To evaluate the machined surface morphologies, the traditional method is to use a series of statistical parameters of the surface contours, represented by the surface roughness [22,23]. However, most of these statistical parameters, like *R*_a_, *R*_q_ for 2D profiles and *S*_a_, *S*_q_ for 3D surfaces, unfortunately, are heavily dependent on the practical measurement conditions (e.g., the resolution of measurement instruments), and are easily affected by the evaluation uncertainty [24], frequency-based errors [25], and measurement noise [26]. Therefore, they could not accurately describe the machined surface quality and texture features [27]. To bridge this gap, a great number of efforts have been made to evaluate machined surface morphologies during the past decades. For instance, the power spectrum density (PSD) [28,29] has been used to extract the frequency information from machined surfaces, while the continuous wavelet method [30] has been employed to analyze the dynamic evolution process of the dominated frequency with the change of machining distance. These methods are all based on the Fast Fourier Transform analysis of the surface contour data, which are similarly affected by the measurement conditions. 

Therefore, finding an intrinsic parameter that is independent of measurement accuracy, to characterize the machined surfaces, has become an essential task. Fractal dimension (*F*_D_) has been acknowledged as one promising approach [31,32,33] and could evaluate the complexity of some objects in anomalous dimensions and provide a deeper insight into the surface generation processes, compared with surface roughness [34]. The fractal dimension is defined as a ratio of the statistical index to the scale at which it is measured [35], thus it could accurately evaluate the complexity of the accessed objects without any disturbance from the measurement resolution. Qu [36] used the fractal dimension to uncover the relationship between the micro-milling parameters of rolled Elgiloy material and the resultant surface quality. It was reported that the feed rate plays an important role in affecting surface complexity. Zheng [37] similarly estimated the surface topography of SiCp/Al machined by UVA grinding based on the fractal theory, and a fitting calibration formula that links the *F*_D_ to *R*_a_ was developed. Chen and Li [30,38] have attempted to adopt fractal dimension to analyze the microscope features (e.g., micro-waviness) on the large-aperture crystal optics fabricated by single-diamond fly-cutting method, but they did not further reveal the effect of brittle- and ductile-regime machining on the fractal features of these fly-cutting surfaces.

The above literature review shows that although the fractal dimension has been identified as a promising approach to estimating the machined surface morphologies comprehensively [30,36,37], little work has been done to use fractal dimension to identify the brittle/ductile micro-milling conditions of KDP crystals, and it is important to evaluate the micro-milled surface qualities and resultant optical performance.

For bridging this research gap, a systematic fractal analysis has been carried out to evaluate the machined surface morphologies of KDP crystals by the micro-milling process. Firstly, a series of micro ball-end milled KDP surfaces was produced with different surface qualities and morphologies. Then, the corresponding three- and two-dimensional fractal dimensions were calculated based on the Box-counting method, respectively, and analyzed by combining the related surface morphologies. The underlying relationship between the brittle/ductile-machined surfaces and their fractal dimension features (i.e., amplitude and symmetry) was explored, which could contribute to improving the repaired surface quality and facilitating the application of the micro-milling repair technique.

## 2. Fractal Analysis Based on the Box-Counting Approach

Fractal has been acknowledged as a promising mathematical approach to analyzing and evaluating the geometrical features which are self-affine, generally adopting fractal dimension as its descriptor. The fractal dimension (*F*_D_) is usually a non-integer when compared with the traditional topological dimension in metrology, which is a quantitative evaluation of the geometrical irregularity of target objects over multiple scales. Many kinds of approaches have been proposed to estimate the fractal dimension, like the Box-counting dimension, Hausdorff dimension, correlation dimension [36,39], and so on. Among them, the Box-counting approach is the most widely used, as it is consistent with the basic definition of fractal theory and is easy to implement [34]. Thus, this method is adopted here for the calculation of the fractal dimension of the three-dimensional (3D) surface and two-dimensional (2D) cross-section contour of the micro-milled KDP surfaces.

**Figure 1:**
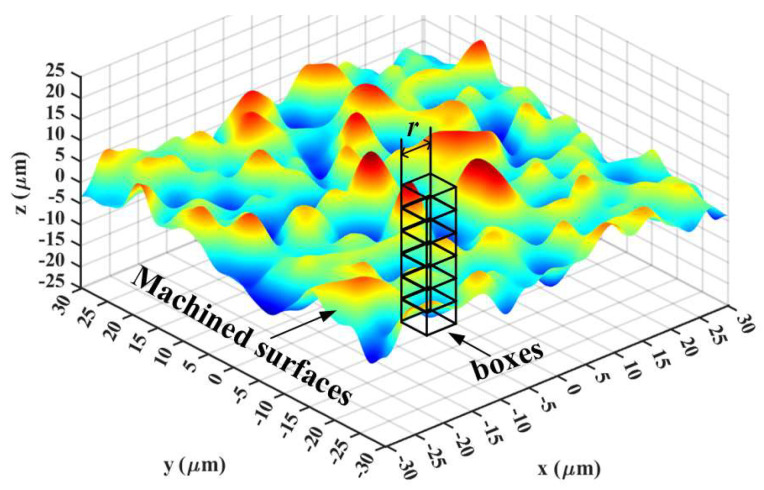
Schematic of fractal dimension calculation using the 3D Box-counting approach.

The 3D Box-counting approach is shown in Figure 1. It is an extension of the simple 2D Box-counting algorithm to high-dimensional space. Its introduction gives a quantitative description of the complex extent of 3D surface topographies. The more complex the shape, the larger the *F*_D_. The 3D *F*_D_ calculation process is demonstrated below:

(1) Use small cubes to envelop the whole surface. These small cubes should have the same side length *l* (*l* is used as the initial observation scale, which can be any scale), and count the cube numbers (*N_l_*) that completely envelop the 3D surface topography.

(2) Analyze the obtained surface data and fractal. If its minimum and maximum height values were in *P*th and *Q*th small cubes, the cube number *n_l_* (*i*, *j*) that can envelop the fractal at the (*i*, *j*)th element should be [34]

(1) $$n_{l}\left(i,j\right)=Q-P+1$$

Moreover, the cube number *N_l_*(i, j) which could fully cover the whole surface is [34]

(2) $$N_{l}\left(i,j\right)=\sum_{i,j}n_{l}\left(i,j\right)$$

(3) Change the value of *l*, and repeat the processes of step (2). The *N_l_* under different measurement scales can be obtained and then fit the data (ln(1/*l*), ln(*N_l_*)) in double logarithmic coordinates. The fitted slope is the 3D *F*_D_ of micro-milled KDP surfaces [33,34]

(3) FD=lnNlln1l

**Figure 2:**
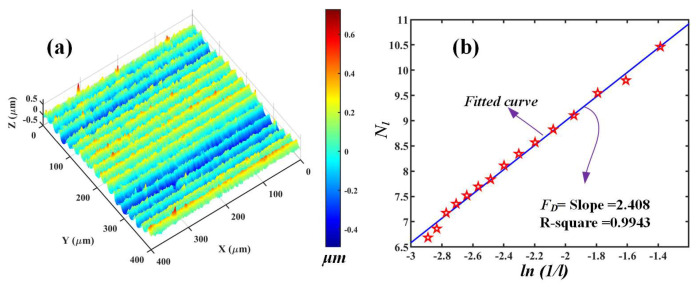
Calculating the fractal dimension (FD) based on the Box-counting method: (**a**) a sample KDP surface produced by micro-milling; (**b**) the calculated fractal dimension of the micro-milled surface in (**a**).

To make the *F*_D_ calculation clear, an example is presented in Figure 2. For a micro-milled surface presented in Figure 2a, the calculation procedures of *F*_D_ could be divided into the following steps. Firstly, a series of cubes with a reasonable size (*l*) are selected and stacked side-by-side to cover the entire 3D surface, as shown in Figure 1. Afterwards, the total number of non-empty boxes *N_l_* required to completely cover the entire object is then obtained. Furthermore, by changing the box size *l*, the same counting process repeats gradually. Finally, the fitted curves of the ln (*N_l_*) versus ln(1/*l*) can be obtained at different box sizes, and the fitted slope is the corresponding *F*_D_ as shown in Figure 2b. The R-square of the fitting is 0.9943, indicating a very high confidence of calculation results. It is worth noting that this study is not focused on developing a novel method to calculate the fractal dimension but rather on attempts to use fractal dimension to identify the generation mechanism (i.e., via brittle or ductile material removal) of machined surfaces, which is the novelty of this manuscript.

## 3. Experimental Design and Details

**Figure 3:**
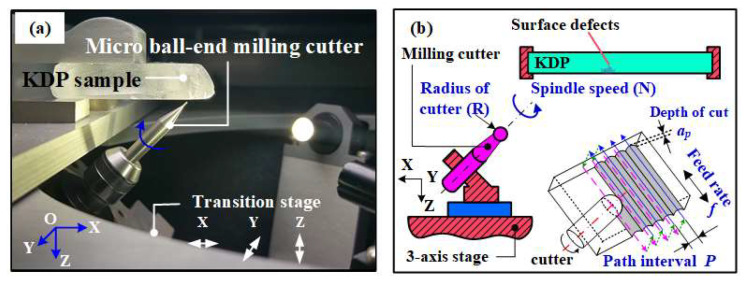
(**a**) Image of micro-milling repair system for KDP [46] and (**b**) the schematic of milling processes with parallel trajectory [21].

To obtain the 3D topography data of the machined surfaces for fractal analysis, a series of micro-milling tests were performed on a house-built precision machine, which is particularly designed for the engineering repair of KDP optical components [40,41]. This machine tool has multi-function, like the vision-based detection of surface defects [42], in-situ monitoring of micro-milling repair processes [43], and automatic tool setting [44]. Regarding the micro-milling repair function, it consists of a three-axis motion platform and a high-speed electric spindle. The platform is a linear motion assembly, the resolution of which along the *X*, *Y*, and *Z* axis is 0.01 µm, 0.01 µm, and 0.05 µm, respectively. The positioning accuracy is smaller than 1 µm for these three axes. Figure 3 shows the schematic of the three-axis ball-end milling system. The spindle is fixed on the motion platform. A micro ball-end milling cutter (SSBL200, NS Tool) was adopted in this test, the radius of which is 0.25 mm. According to our previous study [45], the cutter shank, as well as the spindle, was placed at a 45° inclination angle to KDP surfaces in order to avoid the tip of this spherical cutter engage in the milling process, causing brittle-regime machining.

As shown in Figure 3b, parallel milling paths were adopted here for simulating the actual milling trajectory of the cutter during the actual KDP repair processes. The distance between adjacent trajectories is known as path interval (*P*), which was set as 25 μm here [46]. The spindle speed (*N*) and milling depth (*a_p_*) are 5 × 10^4^ RPM and 2 μm, respectively. The feed per tooth has a dominant role in the brittle-to-ductile transition of material removal behaviors of KDP crystals when the spindle speed and depth of cut have been set as their optimized values (*N* ≥ 5 × 10^4^ RPM, *a_p_* ≤ 2 μm) [47,48]. To better clarify the capacity of fractal dimension in identifying the generation mechanism of machined surfaces (i.e., via brittle or ductile material removal), various machined surfaces with different qualities are required. Thus, feed per tooth has been set in a wide range (i.e.,0.50 µm/z, 1.00 µm/z, 1.50 µm/z, 2.00 µm/z, 2.50 µm/z, and 3.00 µm/z) for producing brittle, brittle-to-ductile, and ductile surfaces, according to our previous study about the effect of feed rate per tooth on the brittle-to-ductile transition in micro-milling processes of KDP crystals [47,48].

After the micro-milling tests, all milled surfaces will be observed by scanning electron microscope (SU8100, Hitachi High-Tech. Co., Ltd., Tokyo, Japan), and white light interference (NewView 3200, Zygo Co., Ltd., Middlefield, CT, USA). Following that, the surface roughness, as well as the three-dimensional data of surface topographies, were obtained. Every machined surface was measured three times. The presented results are the average values of three times the measured results.

## 4. Results and Discussions

### 4.1. Analysis of Micro-Milled KDP Surface Morphologies 

**Figure 4:**
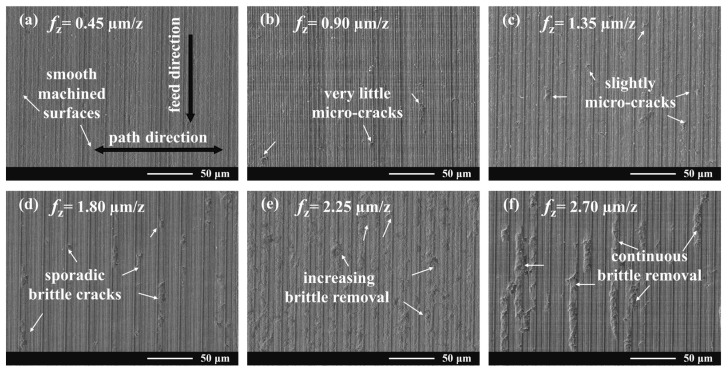
SEM images of micro-milled KDP surface at various feed rates per tooth.

Figure 4 presents the SEM images of the micro-milled KDP surfaces under different feed rates where various material removal modes (i.e., brittle- or ductile-regime mode) might occur. As shown in Figure 4a, a fairly smooth morphology could be observed without any cracks, implying that KDP was milled in ductile-regime mode. As the feed per tooth rises, the actual undeformed chip thickness (UCT) during each cutting process (i.e., every rotation of the milling cutter) also increases synchronously [49]. Once the actual UCT exceeds the critical UCT of brittle-to-ductile transition, micro-cracks will engage in material removal behaviors [49,50] and occur on machined surfaces, as shown in Figure 4b–d. These micro-cracks could prove a mixed material removal mode or ductile-to-brittle transition occurred on these machined surfaces. With the rise of feed per tooth, the actual UCT would further increase, giving the risk of generation of brittle removal (see Figure 4e), which sees an increasing number of brittle cracks on the micro-milled KDP surfaces. Furthermore, the observed surface morphology in Figure 4f is quite dissimilar to those shown in Figure 4a–d. In this case, the obtained surface was fully covered with lots of continuous macro-brittle fractures, indicating that the brittle-regime mode dominated material removal processes.

**Figure 5:**
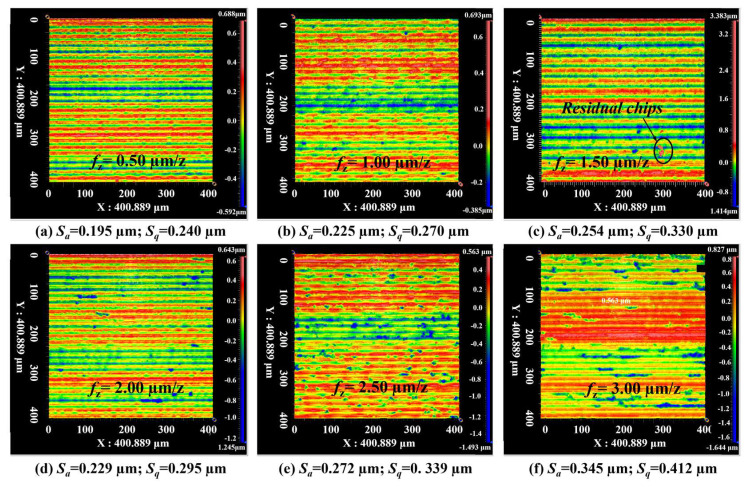
3D morphologies of micro-milled KDP surfaces at various feed rates per tooth.

Meanwhile, Figure 5 shows the surface topographies of micro-milled KDP surfaces observed by the white light interferometer, which could evaluate the surface quality quantitatively (i.e., surface roughness) and provide 3D topography data for fractal analysis. It was found that, when the feed rate per tooth rises gradually, the features (i.e., brittle cracks) on the presented surfaces show a similar increasing trend with those observed by SEM (see Figure 5). This scenario further illustrates the different material removal behaviors engaged in these micro-milling processes. However, it is worth noting that although the material removal behaviors are slightly different on the machined surfaces shown in Figure 5b,d, the measured roughness of these two surfaces is quite close, with a very small deviation of only 0.004 μm. This means that the surface roughness fails to characterize and reflect the subtle change of microscopic texture and structure of these micro-milled KDP surfaces.

### 4.2. 3D Fractal Analysis on the Machined Surface Morphologies 

**Figure 6:**
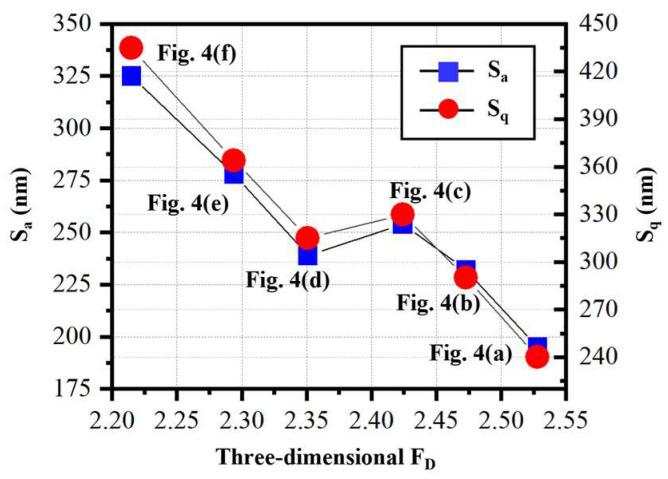
Surface roughness of the micro-milled KDP surfaces versus the calculated 3D fractal dimension.

Based on the above discussion, both the arithmetical mean height (*S*_a_) and root mean square height (*S*_q_) of the measured surfaces, which are the most widely-used surface roughness parameters, could not be able to accurately characterize the topography complexity of the precision micro-milled KDP surfaces. In contrast to *S*_a_ and *S*_q_, the 3D fractal dimension (*F*_D_) has been reported to have a powerful capacity to quantitatively evaluate the topography complexity of the machined optical surfaces [30,38]. As mentioned in Section 2, the values of *F*_D_ are equal to the slopes of ln*N_l_*/ln(1/*l*), so the value variation of calculated *F*_D_, which could be caused by the changes of box numbers required to envelop the surface with the same box size, can accurately characterize the evolution of the complexity of assessed surface topography. To acquire the relationship between *S*_a_, *S*_q_, and *F*_D_, six micro-milled KDP surfaces with different surface quality were analyzed. Figure 6 presents the obtained 3D *F*_D_ of micro-milled KDP surfaces and the corresponding surface roughness (*S*_a_ and *S*_q_).

Meanwhile, to better uncover the correlation between *S*_a_, *S*_q_, and *F*_D_, a correlation analysis was performed between these values with two outcomes: correlation coefficient, *R,* and statistical significance, *P*. The calculated *R* can be used to indicate the extent of the correlation between two assessed objects. The *p*-value indicates the probability, by random chance, of obtaining a correlation as large as the observed values. The detailed formula for performing correlation analysis can be expressed below:

(4) $$[R,\;P]=\frac{\sum_{i=1}^{l}\left(S_{i}-\bar{S}\right)\left(F_{i}-\bar{F}\right)}{\sqrt{\sum_{i=1}^{l}{\left(S_{i}-\bar{S}\right)}^{2}{\left(F_{i}-\bar{F}\right)}^{2}}}$$

where *S* is the measured surface roughness (i.e., *S*_a_, *S*_q_) while *F* is the calculated fractal dimension (*F*_D_). The correlation coefficient *R* is in the range of [–1, 1]. If *R* is positive, the accessed objects are positively correlated, while negative R-values mean the accessed objects are negatively correlated. The size in the absolute value of *R* means the correlation extent. Besides, if the calculated *p*-value is less than 5%, the correlation between the variables is usually considered statistically significant in the results.

Table 1 lists the calculated correlation results between the 3D *F*_D_ and *S*_a_, *S*_q_. By observing *R*- and *p*-values, it was revealed that the calculated *F*_D_ has a statistically significant correlation with the measured *S*_a_ and *S*_q_. Taking *S*_a_ for instance, the coefficient *R =* −0.8985 sees a negative correlation between *S*_a_ and *F*_D_ with a small *p*-value (0.0149 < 0.05). This means, although the correlation between *F*_D_ and *S*_a_ is not strict, the *F*_D_ overall reduces as *S*_a_ rises. The evolution of *S*_q_ versus *F*_D_ keeps like that of *S*_a_. Thus, if the machined surface quality was observed in view of 3D fractal dimension, it can be found that, the larger the *F*_D_, the finer the microscopic morphology of machined KDP surfaces, and the finer the machined surface textures, indicating the better micro-milled surface quality produced. When it comes to the surface with smaller *F*_D_, a great number of micro-cracks and brittle removal take place, as shown in Figure 5, implying that a worse surface quality with coarse textures was produced. Furthermore, as mentioned above, the two machined KDP surfaces shown in Figure 5b,d have similar *S*_a_, although their surface quality and surface textures are quite different. However, combining the surface morphologies shown in Figure 5b,d, the calculated fractal dimension presented in Figure 6 demonstrates that the micro-milled surface with a larger *F*_D_ value has a better surface quality. Thus, the fractal dimension could be employed here as a powerful approach to quantitatively evaluate the micro ball-end milled KDP surfaces and to identify the brittle or ductile machining behaviors.

### 4.3. 2D Fractal Analysis on the Machined Surface Morphologies

#### 4.3.1. Anisotropy Analysis of the Micro Ball-End Milled Surfaces

Through the above analysis, the 3D *F*_D_ is good enough for characterizing the overall quality of the machined surfaces, as it treats them as a whole during the calculation processes. This indicates that the 3D *F*_D_ loses the ability to characterize local texture features of the machined surfaces, especially for anisotropic surfaces like milled surfaces. During the micro-milling processes, the milling cutter will feed not only along its feeding direction but also move perpendicular to its feeding direction (i.e., path direction) with parallel trajectories, resulting in different types of residual tool marks [45,46]. To achieve ductile machining for KDP, the federate per tooth is selected as about several μm close to its critical UCT of brittle-to-ductile transition [51], and is much smaller than the path interval (25 μm) between adjacent trajectories. This scenario inevitably causes the residual height of tool marks along the path direction to be much larger than that along the feed direction, further forming anisotropic features on the machined surfaces. Besides, this kind of anisotropic feature also means the contours of cross-sectional surfaces along different directions have different characteristics, which could be used for calculating the 2D fractal dimension. Thus, the calculated 2D along different directions could be used to characterize the anisotropy of the micro ball-end milled surfaces.

**Figure 7:**
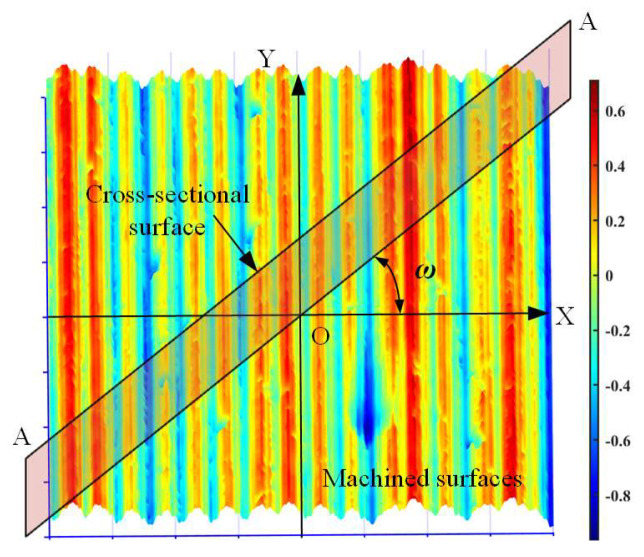
Schematic of the cross-sectional surface contours (A-A) used for calculating the 2D fractal dimension.

When calculating the 2D *F*_D_, as shown in Figure 7, the center of the obtained surface could be regarded as the coordinate origin. The *X*-axis direction in the measurement coordinate system is assigned as the direction with the angle of *θ* = 0°, while the *Y*-axis direction in the coordinate system is assigned as the direction with the angle of *θ* = 90°. The plane passing through the coordinate origin and perpendicular to the machined surface can be defined as the cross-section plane. The included angle between this cross-section plane and the *X*-axis direction could vary from 0° to 360°. The interaction contour between the cross-section plane and machined surface is the cross-sectional surface profile, which can be used as the sample data for the 2D fractal dimension. Thus, the 2D fractal dimension might be varied even for the same machined surface because the adopted cross-sectional surface profile might be selected with different included angles. Therefore, if the direction of cross-sectional surface profiles could be evenly selected from the entire circumference, the circumferential fractal dimension could be obtained.

**Figure 8:**
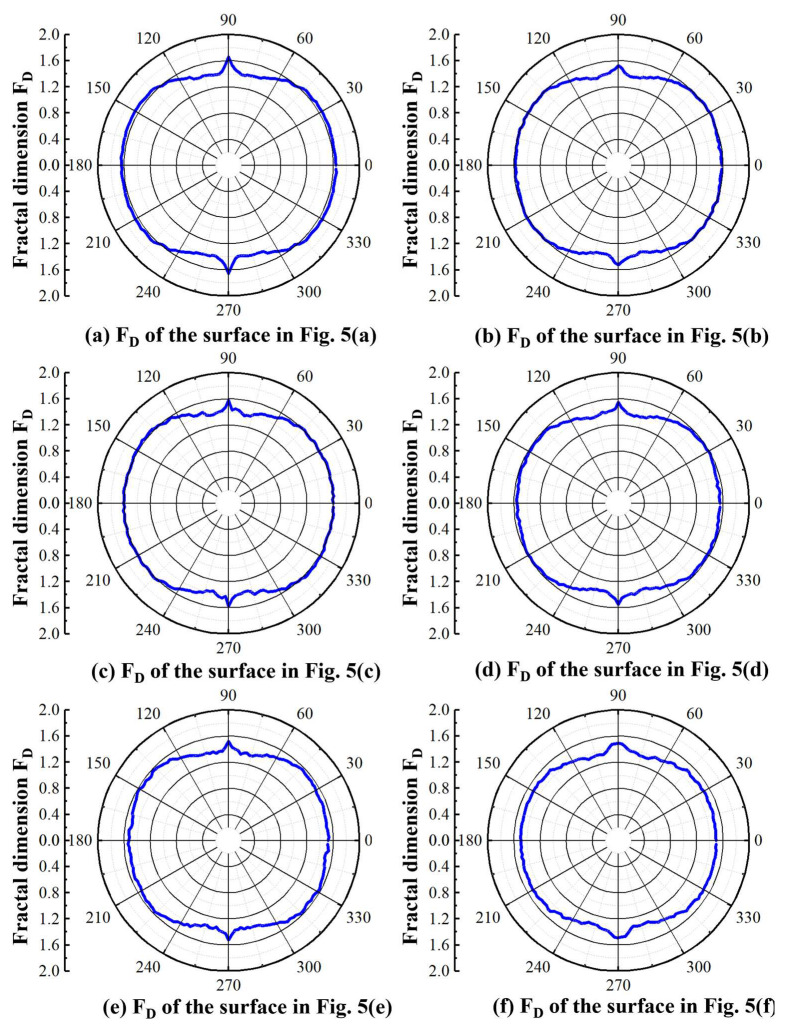
The circumferential 2D fractal dimension of micro-milled KDP surfaces processed with different milling parameters.

Figure 8 presents the circumferential 2D *F*_D_ of the micro-milled KDP surfaces processed with different milling parameters. The anisotropic features of these surfaces can be observed more visible and intuitively. The maximum values of each circumferential *F*_D_ occur at the angle of 90°, which corresponds to the feed direction. It is worth noting that the values of circumferential *F*_D_ at both sides of the *Y*-axis tend to decrease substantially while the *F*_D_ values around *X*-axis keep stable, which further quantitatively proves the existence of anisotropic feature on machined KDP surfaces. Besides, although the shapes of these six circumferential *F*_D_ shown in Figure 8 are similar, the overall amplitude of their values is slightly different. For example, the *F*_D_ in Figure 8a is higher than 1.6 while those in Figure 8f are less than 1.6, indicating the different surface quality and textures on these machined surfaces. 

**Figure 9:**
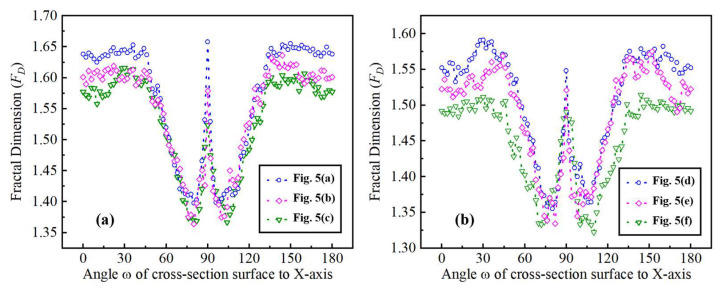
The 2D fractal dimension at various included angles to the *x*-axis on the different machined KDP surfaces, as shown in Figure 5.

To better compare the evolution of surface textures with the change of included angles, the 2D *F*_D_ values of these six micro-milled surfaces (see in Figure 4 and Figure 5) were presented in the cartesian coordinate system with the included angle range of 0°~180°, as shown in Figure 9. It is noteworthy that the *F*_D_ profiles at 0°~180° are distributed symmetrically with those at 180°–360° (see in Figure 8). Thus, only the *F*_D_ profiles at 0°~180° were selected and presented in Figure 9. For the surface shown in Figure 5a, it can be seen that the *F*_D_ amplitudes tend to be stable at around 1.64 within the included angle range of 0°~45°, as shown in Figure 9a, and then fall off quickly with the rise of included angles, reaching a low point of 1.40 at around 80°. Afterward, the *F*_D_ values show a sharp increasing trend and peak at their maximum close to 1.67 at 90° (i.e., the feed direction), followed by a quick drop back to 1.40 at 100°. With the further increase of included angles, the *F*_D_ values rebound noticeably and subsequently level out at 1.64 when the angles become higher than 135°. The *F*_D_ profiles of the surfaces shown in Figure 5b,c demonstrate a similar evolution tendency, except that their overall amplitudes are slightly smaller than those of Figure 5a. These phenomena clearly show the strong symmetry of the 2D fractal dimension and further clarify the anisotropy of these micro ball-end milled KDP surfaces which are normally produced by ductile-regime removal mode.

When it comes to the surfaces shown in Figure 5d–f, the overall changing trend of their 2D *F*_D_ profiles is similar to those in Figure 5a–c with the rise of included angles to the *X*-axis. As seen in Figure 9b, one noticeable difference is that the *F*_D_ values of the cross-sectional surface fluctuate markedly and considerably. For example, the *F*_D_ values of Figure 5e surfaces within the angle range of 0°~45° and 135°~180° are not as stable as those of Figure 5a. This scenario should be attributed to the random generation of brittle micro-cracks on these machined KDP surfaces as observed above from Figure 4. Furthermore, the symmetry of the 2D fractal dimension with *w* = 90°, as the symmetry axis also becomes weak due to fluctuations of *F*_D_ values at different angles, indicating that these surfaces have a relatively weaker anisotropic feature owing to the occurrence of brittle-regime removal behaviors.

Thus, through the above analysis, it can be concluded that the extents of the anisotropy of the micro-milled KDP surfaces severely depend on the material removal behaviors (i.e., ductile, or brittle removal modes), suggesting that the 2D *F*_D_ of cross-sectional surfaces could be utilized to distinct the ductile-regime removal from brittle-regime removal in micro-milling processes of KDP crystals.

#### 4.3.2. Fractal Dimension Analysis of Cross-Section Surface Contours

**Figure 10:**
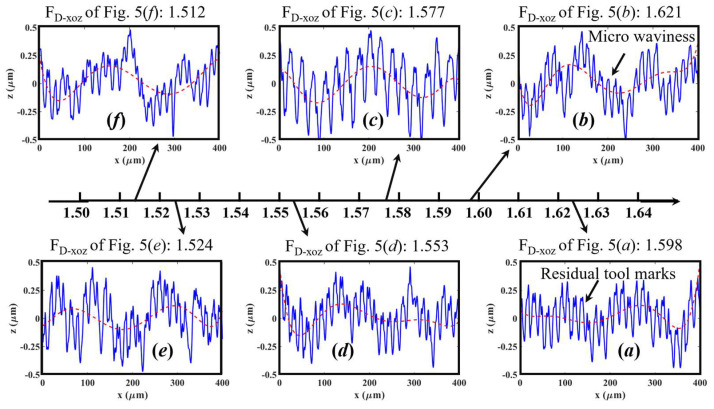
The cross-sectional contours of KDP surfaces shown in Figure 5 perpendicular to the feed direction and the corresponding 2D fractal dimension (*F*_D_). The red dash lines are the fitted curves through cubic polynomials.

**Figure 11:**
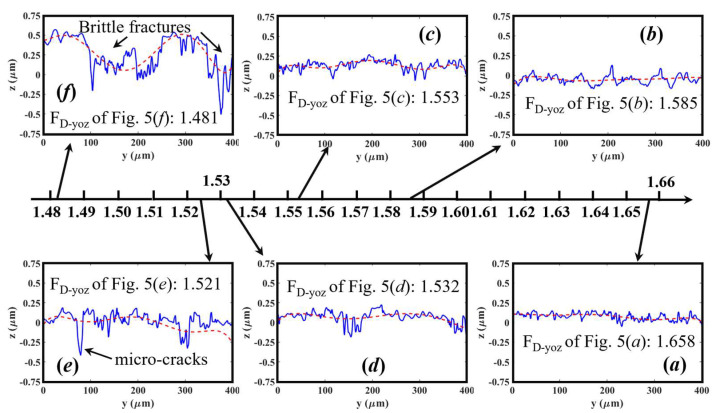
The cross-sectional contours of KDP surfaces shown in Figure 5 parallel with the feed direction and the corresponding 2D fractal dimension (*F_D_*). The red dash lines are the fitted curves through cubic polynomials.

As revealed in Section 4.3.1, the *F*_D_ values of the cross-sectional surface contours and their distribution could be employed to identify the various material removal behaviors. To further uncover the underlying relationship between the 2D *F*_D_ and brittle/ductile material removal modes, two types of cross-sectional surface contours on each machined surface in Figure 5 were selected: one is perpendicular to the milling feed direction (i.e., in the XOZ-plane) while the other one is in parallel with the milling feed direction (i.e., in the YOZ-plane). Then, the corresponding 2D fractal dimensions (*F*_D_-_XOZ_ and *F*_D_-_YOZ_) were calculated and are presented in Figure 10 and Figure 11, respectively.

One can see that the six cross-sectional surface contours along the path direction are similar in structures (see Figure 10). To further understand their structural characteristics, cubic polynomials were used to fit these surface contours. The fitted curves are presented by red-dash lines in Figure 10 and show obvious waviness features. Thus, it was found that these surface contours along the path direction mainly consist of residual tool marks and micro-waviness due to the dynamic response between the micro-milling cutter and workpiece [52]. As they are similar in structure, it seems to be hard to identify the particular material removal modes through the cross-sectional surface contours along this path direction.

In contrast to them, the cross-sectional surface contours in parallel with the feed direction, as shown in Figure 11, show a significant difference, and the corresponding fractal dimension *F*_D_ related to ductile-regime modes are prone to be higher than those related to brittle-regime modes. When the 2D *F*_D_ increases from 1.481 to 1.658, it witnesses a significant change of surface contours from rough to fine and smooth, as well as a transition from brittle- to ductile-regime removal. To be specific, as depicted in Figure 11f, due to the occurrence of brittle cracks and fractures, the cross-sectional surface contours are quite rough with many large peak-valley fluctuations, and the corresponding *F*_D_-_YOZ_ is the smallest (1.481). A similar phenomenon can be found in Figure 11d,e, where the brittle cracks have a negative impact on their fractal dimension. However, when the surface contours become smooth without obvious brittle cracks, as shown in Figure 11a, the 2D *F*_D_ achieved its maximum (*F*_D_-_YOZ_ = 1.658). For the surface contours shown in Figure 11b,c, there are only a few fluctuations, so their *F*_D_ is just between 1.532 and 1.658.

Thus, by comparing the contents presented in Figure 10 and Figure 11, it was found that the cross-sectional surface contours generated by brittle- or ductile-regime are much different in the cutter feeding direction. This is because the surface contours along the feed direction normally have smaller residual tool marks and thus are much easier to highlight the existence of brittle cracks and fractures, compared with the surface contours perpendicular to the feed direction with higher residual-height tool marks. 

## 5. Conclusions

In this work, the fractal dimension has been introduced to characterize the surface morphologies of soft-brittle KDP crystals produced by micro bell-end milling processes, as the conventional method (i.e., surface roughness) can only give the machined surface an overall estimation and fails to characterize the texture features of surface morphologies. The main conclusions are summarized below:

(1) The 3D machined surfaces and their typical 2D cross-sectional contours were analyzed using the Box-counting method for calculating corresponding 3D and 2D fractal dimensions, respectively. The calculated fractal dimension of different micro-milled KDP surfaces was discussed comprehensively, combining the analysis of the surface quality and textures. It was found that there was a negative correlation between the 3D fractal dimension and surface roughness (*S*_a_ and *S*_q_). This means that the worse the surface quality, the smaller the fractal dimension.
(2) The circumstances 2D fractal dimension of cross-sectional surfaces has been approved to quantitively characterize the anisotropy of the micro ball-end milled surfaces, which could not be analyzed by surface roughness. If the 2D fractal dimension is distributed symmetrically, the surface contours are supposed to be generated by ductile-regime removal. While it is distributed asymmetrically, the surface contours should be occupied by brittle cracks and fractures and corresponding machining processes in brittle-regime. This phenomenon becomes more significant for the cross-sectional surface contours along the feed direction than those perpendicular to the feed direction.

In all, the micro ball-end milled KDP surfaces could be characterized comprehensively by systematical fractal analysis (i.e., 3D and 2D fractal dimensions), and the fractal dimension with high values tends to occur on the high-quality smooth surfaces, which are normally produced by ductile-regime machining processes.

## Figures and Tables

**Table 1:** Calculated correlation between 3D fractal dimension and surface roughness.

Ex. No	*S*_a_	*S*_q_	*F*_D_	*R*	*p*-Value
1	195	240	2.528	−0.8985 (*S*_a_ vs. *F*_D_)	0.0149 (*S*_a_ vs. *F*_D_)
2	225	270	2.473		
3	254	330	2.424		
4	229	295	2.351	−0.9117 (*S*_q_ vs. *F*_D_)	0.0114 (*S*_q_ vs. *F*_D_)
5	272	339	2.294		
6	345	412	2.215		

## Data Availability

Not applicable.

## References

[1] Chen M., Ding W., Cheng J., Yang H., Liu Q. (2020). Recent Advances in Laser-Induced Surface Damage of KH2PO4 Crystal. Appl. Sci..

[2] Liu Q., Chen M., Liao Z., Feng J., Xu D., Cheng J. (2021). On the improvement of the ductile removal ability of brittle KDP crystal via temperature effect. Ceram. Int..

[3] Cheng J., Yang H., Liu Q., Zhao L., Liu Z., Liu H., Wang T., Xiao Y., Hu K., Chen M. (2019). Characterization of manufacturing-induced surface scratches and their effect on laser damage resistance performance of diamond fly-cut KDP crystal. Results Phys..

[4] Liu Q., Liao Z., Axinte D. (2020). Temperature effect on the material removal mechanism of soft-brittle crystals at nano/micron scale. Int. J. Mach. Tools Manuf..

[5] Yang H., Cheng J., Liu Z., Liu Q., Zhao L., Tan C., Wang J., Chen M. (2020). Secondary peak of downstream light field modulation caused by Gaussian mitigation pits on the rear KDP surface. Opt. Express.

[6] Liu Z.C., Geng F., Li Y.G., Cheng J., Yang H., Zheng Y., Wang J., Xu Q. (2018). Study of morphological feature and mechanism of potassium dihydrogen phosphate surface damage under a 351 nm nanosecond laser. Appl. Opt..

[7] Tan C., Zhao L., Chen M., Cheng J., Yang H., Liu Q., Yin Z., Liao W. (2021). Formation mechanism of surface morphology in the process of CO2 pulsed laser processing of fused silica optics. Opt. Laser Technol..

[8] Lawrence W.H., Raymond M.B., Walter G., Mary A.N., Eugene E.D., William A.M., Samuel L.T., Steven R.S., Pamela K.W., Michael Douglas S. Methods for Mitigating Growth of Laser-Initiated Surface Damage on DKDP Optics at 351 nm. Proceedings of the Laser-Induced Damage in Optical Materials: 2002 and 7th International Workshop on Laser Beam and Optics Characterization.

[9] Lei H., Cheng J., Yang D., Zhao L., Chen M., Wang J., Liu Q., Ding W., Chen G. (2022). Effect of Pre-Existing Micro-Defects on Cutting Force and Machined Surface Quality Involved in the Ball-End Milling Repairing of Flawed KDP Crystal Surfaces. Materials.

[10] Cheng J., Xiao Y., Liu Q., Yang H., Zhao L., Chen M., Tan J., Liao W., Chen J., Yuan X. (2018). Effect of surface scallop tool marks generated in micro-milling repairing process on the optical performance of potassium dihydrogen phosphate crystal. Mater. Des..

[11] Yang S., Zhang L., Wu Z. (2021). Effect of Anisotropy of Potassium Dihydrogen Phosphate Crystals on Its Deformation Mechanisms Subjected to Nanoindentation. ACS Appl Mater Interfaces.

[12] Borc J., Sangwal K., Pritula I., Dolzhenkova E. (2017). Investigation of pop-in events and indentation size effect on the (001) and (100) faces of KDP crystals by nanoindentation deformation. Mater. Sci. Eng. A.

[13] Yang S., Zhang L. (2021). Characterization of mechanical properties and failure of potassium dihydrogen phosphate under mechanical stressing. Ceram. Int..

[14] Li C., Zhang Y., Zhou G., Wei Z., Zhang L. (2020). Theoretical modelling of brittle-to-ductile transition load of KDP crystals on (001) plane during nanoindentation and nanoscratch tests. J. Mater. Res. Technol..

[15] Li C., Piao Y., Hu Y., Wei Z., Li L., Zhang F. (2021). Modelling and experimental investigation of temperature field during fly-cutting of KDP crystals. Int. J. Mech. Sci..

[16] La Monaca A., Murray J.W., Liao Z., Speidel A., Robles-Linares J.A., Axinte D.A., Hardy M.C., Clare A.T. (2021). Surface integrity in metal machining—Part II: Functional performance. Int. J. Mach. Tools Manuf..

[17] Liao Z., La Monaca A., Murray J., Speidel A., Ushmaev D., Clare A., Axinte D., M’Saoubi R. (2021). Surface integrity in metal machining—Part I: Fundamentals of surface characteristics and formation mechanisms. Int. J. Mach. Tools Manuf..

[18] La Monaca A., Axinte D.A., Liao Z., M’Saoubi R., Hardy M.C. (2021). Towards understanding the thermal history of microstructural surface deformation when cutting a next generation powder metallurgy nickel-base superalloy. Int. J. Mach. Tools Manuf..

[19] Paul G., Carr W., Draggoo V., Hackel R., Mailhiot C., Norton M. (2007). Surface Damage Growth Mitigation on KDP/DKDP Optics Using Single-Crystal Diamond Micro-Machining Ball End Mill Contouring. Laser-Induced Damage in Optical Materials: 2006.

[20] Liu Q., Cheng J., Liao Z., Yang H., Zhao L., Chen M. (2019). Incident laser modulation by tool marks on micro-milled KDP crystal surface: Numerical simulation and experimental verification. Opt. Laser Technol..

[21] Liu Q., Cheng J., Liao Z., Luo X., Yang Y., Li M., Yang H., Tan C., Wang G., Ding W. (2023). Research on the light intensity modulation and characterizing methods of surface texture on KDP optics generated in fly-cutting and micro ball-end milling processes. CIRP J. Manuf. Sci. Technol..

[22] Zeng Q., Qin Y., Chang W., Luo X. (2018). Correlating and evaluating the functionality-related properties with surface texture parameters and specific characteristics of machined components. Int. J. Mech. Sci..

[23] Pan Y., Zhou P., Yan Y., Agrawal A., Wang Y., Guo D., Goel S. (2021). New insights into the methods for predicting ground surface roughness in the age of digitalisation. Precis. Eng..

[24] Haitjema H. (2015). Uncertainty in measurement of surface topography. Surf. Topogr. Metrol. Prop..

[25] Podulka P. (2022). Selection of Methods of Surface Texture Characterisation for Reduction of the Frequency-Based Errors in the Measurement and Data Analysis Processes. Sensors.

[26] Giusca C.L., Claverley J.D., Sun W., Leach R.K., Helmli F., Chavigner M.P.J. (2014). Practical estimation of measurement noise and flatness deviation on focus variation microscopes. CIRP Ann..

[27] Goodhand M.N., Walton K., Blunt L., Lung H.W., Miller R.J., Marsden R. (2016). The Limitations of Using “Ra” to Describe Surface Roughness. J. Turbomach..

[28] Kuang L., Pang Q., Chen M., Ma L., Xu Y. (2020). Research on influence of cutting parameters on frequency characteristics of KDP surface topography. Qiangjiguang Yu Lizishu/High Power Laser Part. Beams.

[29] Pang Q., Kuang L., Xu Y. (2020). The influence of cutting parameters on micro-topography of frequency features extracted from the machined KH2PO4 surfaces. Proc. Inst. Mech. Eng. Part B: J. Eng. Manuf..

[30] Chen M., Li M., Cheng J., Xiao Y., Pang Q. (2013). Study on the optical performance and characterization method of texture on KH2PO4 surface processed by single point diamond turning. Appl. Surf. Sci..

[31] Mandelbrot B.B., Passoja D.E., Paullay A.J. (1984). Fractal character of fracture surfaces of metals. Nature.

[32] Yadav R.P., Kumar T., Mittal A.K., Dwivedi S., Kanjilal D. (2015). Fractal characterization of the silicon surfaces produced by ion beam irradiation of varying fluences. Appl. Surf. Sci..

[33] Wen T., Cheong K.H. (2021). The fractal dimension of complex networks: A review. Inf. Fusion.

[34] Nayak S.R., Mishra J., Palai G. (2019). Analysing roughness of surface through fractal dimension: A review. Image Vis. Comput..

[35] Wang L.E.I., Luo R., Zhang W.E.I., Jin M., Tang S. (2021). Effects of Fineness and Content of Phosphorus Slag on Cement Hydration, Permeability, Pore Structure and Fractal Dimension of Concrete. Fractals.

[36] Qu D., Wang B., Peng Z. (2017). The influence of processing parameters on surface characteristics in micro-milling thin-walled slot on Elgiloy. Int. J. Adv. Manuf. Technol..

[37] Zheng W., Zhou M., Zhou L. (2017). Influence of process parameters on surface topography in ultrasonic vibration- assisted end grinding of SiCp/Al composites. Int. J. Adv. Manuf. Technol..

[38] Chen M., Pang Q., Wang J., Cheng K. (2008). Analysis of 3D microtopography in machined KDP crystal surfaces based on fractal and wavelet methods. Int. J. Mach. Tools Manuf..

[39] Panigrahy C., Seal A., Mahato N.K., Bhattacharjee D. (2019). Differential box counting methods for estimating fractal dimension of gray-scale images: A survey. Chaos Solitons Fractals.

[40] Yang H., Cheng J., Chen M., Wang J., Liu Z., An C., Zheng Y., Hu K., Liu Q. (2017). Optimization of morphological parameters for mitigation pits on rear KDP surface: Experiments and numerical modeling. Opt Express.

[41] Yang H., Cheng J., Liu Z., Liu Q., Zhao L., Tan C., Wang J., Chen M. (2020). Model Development for Nanosecond Laser-Induced Damage Caused by Manufacturing-Induced Defects on Potassium Dihydrogen Phosphate Crystals. ACS Omega.

[42] Zhao L., Yin Z., Zhang D., Cheng J., Yang H., Tan C., Liu Q., Chen M., Yuan X. (2022). A Novel Subpixel Size Calibration Method for the Size Detection of Microtarget on Large-Aperture Optics Surface. IEEE Trans. Instrum. Meas..

[43] Liu Q., Cheng J., Xiao Y., Yang H., Chen M. (2018). Effect of milling modes on surface integrity of KDP crystal processed by micro ball-end milling. Procedia CIRP.

[44] Zhao L., Cheng J., Yin Z., Yang H., Chen M., Yuan X. (2021). Research on precision automatic tool setting technology for KDP crystal surface damage mitigation based on machine vision. J. Manuf. Process..

[45] Liu Q., Cheng J., Xiao Y., Chen M., Yang H., Wang J. (2018). Effect of tool inclination on surface quality of KDP crystal processed by micro ball-end milling. Int. J. Adv. Manuf. Technol..

[46] Liu Q., Cheng J., Yang H., Xu Y., Zhao L., Tan C., Chen M. (2019). Modeling of residual tool mark formation and its influence on the optical performance of KH2PO4 optics repaired by micro-milling. Opt. Mater. Express.

[47] Chen N., Chen M., Wu C., Pei X. (2017). Cutting surface quality analysis in micro ball end-milling of KDP crystal considering size effect and minimum undeformed chip thickness. Precis. Eng..

[48] Chen N., Chen M., Wu C., Pei X., Qian J., Reynaerts D. (2017). Research in minimum undeformed chip thickness and size effect in micro end-milling of potassium dihydrogen phosphate crystal. Int. J. Mech. Sci..

[49] Fang F., Lai M., Wang J., Luo X., Yan J., Yan Y. (2022). Nanometric cutting: Mechanisms, practices and future perspectives. Int. J. Mach. Tools Manuf..

[50] Axinte D., Huang H., Yan J., Liao Z. (2022). What micro-mechanical testing can reveal about machining processes. Int. J. Mach. Tools Manuf..

[51] Liu Q., Liao Z., Cheng J., Xu D., Chen M. (2021). Mechanism of chip formation and surface-defects in orthogonal cutting of soft-brittle potassium dihydrogen phosphate crystals. Mater. Des..

[52] Chen W., Xie W., Huo D., Yang K. (2018). A novel 3D surface generation model for micro milling based on homogeneous matrix transformation and dynamic regenerative effect. Int. J. Mech. Sci..

